# Phoniatry: otorhinolaryngology expands its limits^☆^

**DOI:** 10.1016/j.bjorl.2015.08.004

**Published:** 2015-09-07

**Authors:** Berenice Dias Ramos

**Affiliations:** a Preceptor of Pediatric Otorhinolaryngology and Phoniatry, Pontifícia Universidade Católica do Rio Grande do Sul (PUC-RS), Porto Alegre, RS, Brazil; b Master degree in Otorhinolaryngology, Universidade Federal de São Paulo (UNIFESP), São Paulo, SP, Brazil; c Professor of Audiology and Auditory Processing, Extensive Training Course in Phoniatry, Brazilian Association of Otorhinolaryngology and Neck and Facial Surgery (ABORL-CCF), São Paulo, SP, Brazil

Several areas of human knowledge are evolving rapidly. Otorhinolaryngology is one of them: endoscopic surgery, cochlear implants, and robotic surgery are now a reality.

Until a few years ago, we faced difficulties obtaining an early diagnosis and treating infants born with hearing impairment (HI). Diagnostic advances, including otoacoustic emission, auditory brainstem response (ABR), steady-state potentials – as well as therapeutic advances with digital amplification devices, cochlear implants, implantable prostheses, etc. – have completely changed the prognosis for children with HI. Nowadays, thanks to technological advances, we can guarantee a completely normal life for these infants.

The main contemporary challenges for professionals working with children exhibiting language delay are related to diseases that have no available laboratory tests to establish the diagnosis and no surgical or clinical treatment to change its evolution. The prognosis depends on establishing the diagnosis as soon as possible and on the right specific speech therapy. In this scenario, the two most significant diseases are autistic spectrum disorder (ASD) and specific language disorder (SLD).

Upon learning of this subject, perhaps many otorhinolaryngologists wonder: “Where are these children, for I do not see them?” Certainly, they are being seen in these same professionals’ offices, because these are highly prevalent diseases, although they are not being diagnosed.

The various disciplines studying language development in children – such as speech therapy, developmental pediatrics, psychiatry, and pediatric neurology – are finding an increased incidence of ASD. The 2014 report of CDC (Center for Disease Control) relates an ASD incidence of 1:68 for 8-year-old children, five times more frequent in males (1:42) than in females (1:189).^1^ Several genetic and epigenetic studies are being conducted to try to unravel the etiology of ASD. Genome sequencing projects showed that there are hundreds of ASD-associated genes.

Regarding SLD, the NIH (National Institutes of Health) states that this disease affects about 7–8% of preschool children and that in 50–70% of cases there is at least one relative suffering the same disturbance.^2^ The correct diagnosis of SLD followed by adequate speech therapy minimizes its consequences, which include reading and writing disorders.

In Brazil, no epidemiological studies on ASD and SLD have been conducted, but it is possible that the Brazilian rates are similar to those of the United States.

Hearing represents the world's largest information gateway. But having normal hearing thresholds is not enough: we need to process and recognize the auditory information in a quick and correct way.^3^ In today's world, where quick thinkers are more valued, it is critical to identify and treat any disease that can interfere with auditory processing. The incidence of otitis media, one of the main causes of auditory processing disorder, increased when there was a dramatic increase in women entering the labor market and placing their infants in nurseries where they encountered an increase in the number of airway infections.

Through electrophysiological tests, such as ABR with speech stimuli, we can identify children with auditory processing disorder which may present reading and writing learning difficulties in their early years of life.^4^

In most cases, patients with language delay or learning disability are initially seen by an otorhinolaryngologist for suspected HI.

What should be the role of the otorhinolaryngologist in these cases? Every time we see a child with language delay and with normal hearing, we must think that, by simply stating that the exam is normal, that everything is fine, and that the child should talk soon, we may be delaying the correct diagnosis and losing a few months of great neuroplasticity.

A more thorough assessment, followed by a proper guidance, can dramatically change the future of a child.

Similarly, it is important to investigate the auditory processing of those children with learning disabilities, for without an accurate diagnosis, they can waste years with inadequate and ineffective therapies.

For many years, otolaryngologists treated hearing problems in the cochlea. But thanks to ABR, hearing also can be assessed in the brain stem. Today, we must be prepared to assess hearing in the brain. Mismatch Negativity (MMN), P300, ABR with speech stimuli, and behavioral assessments of auditory processing offer much information that can assist in the diagnosis and therapeutic management of patients with oral or written language disorders and normal hearing thresholds.

For those who are interested in these topics, the ABORL has been offering the Extensive Training Course in Phoniatry for two years.

## Conflicts of interest

The author declares no conflicts of interest.
